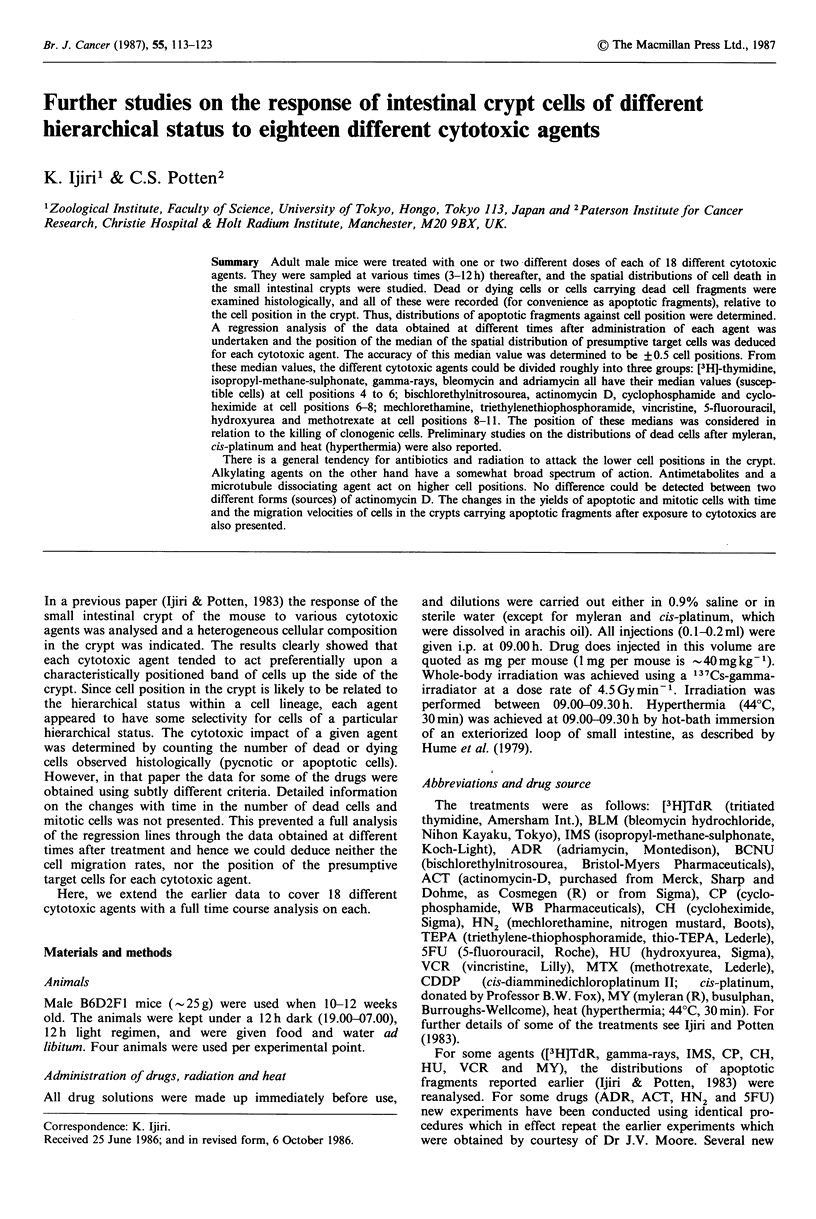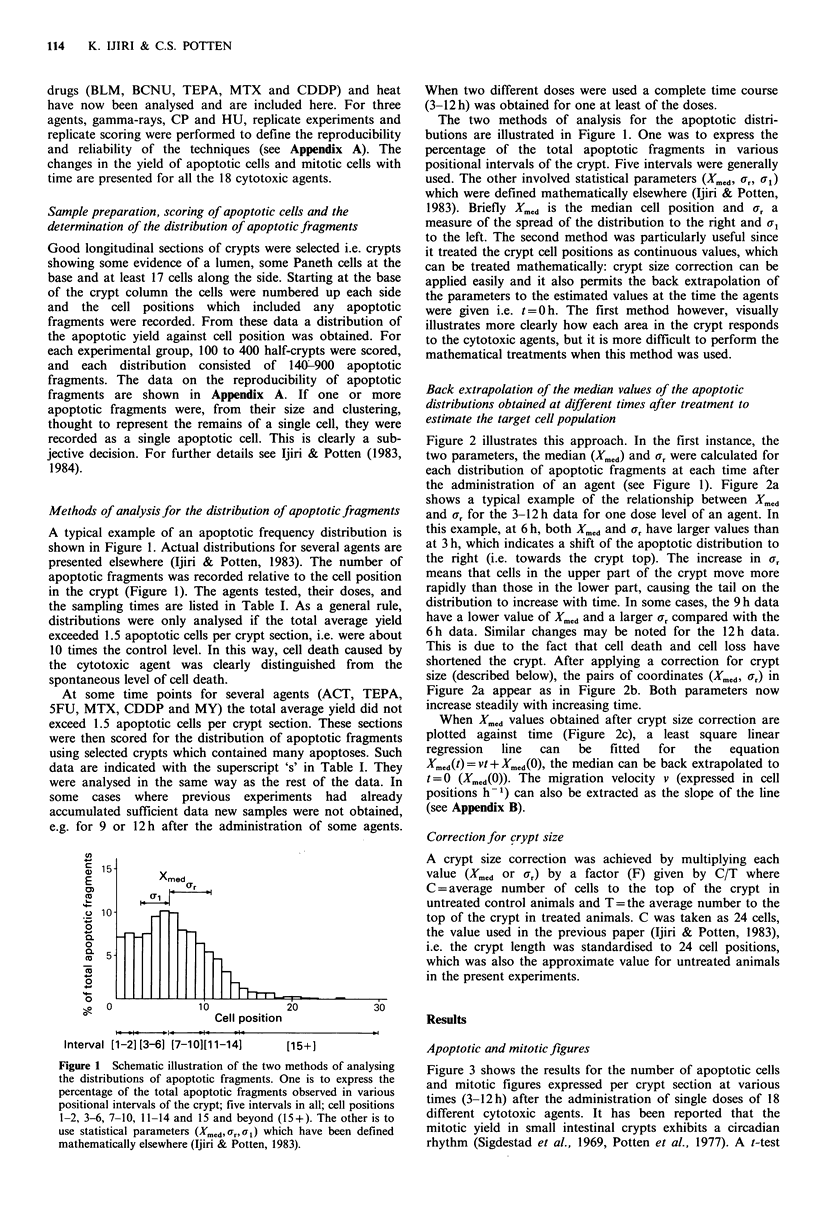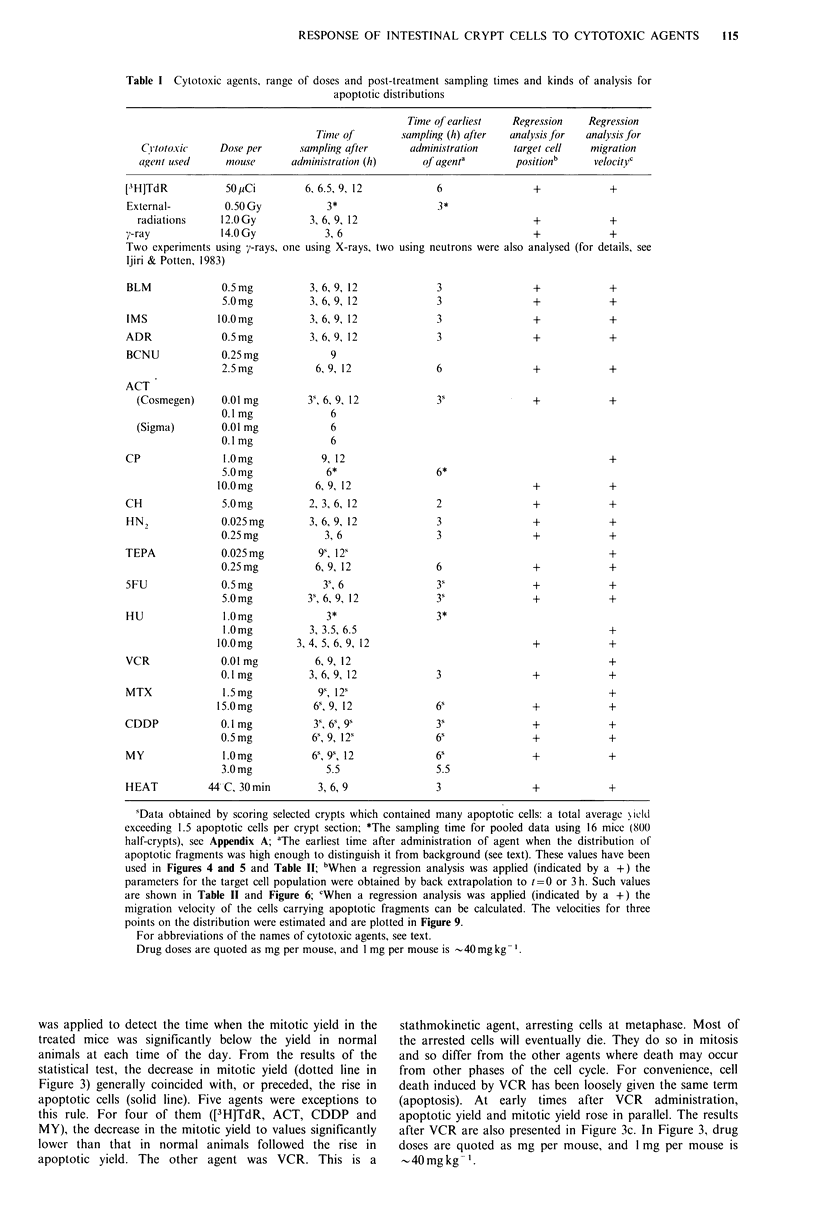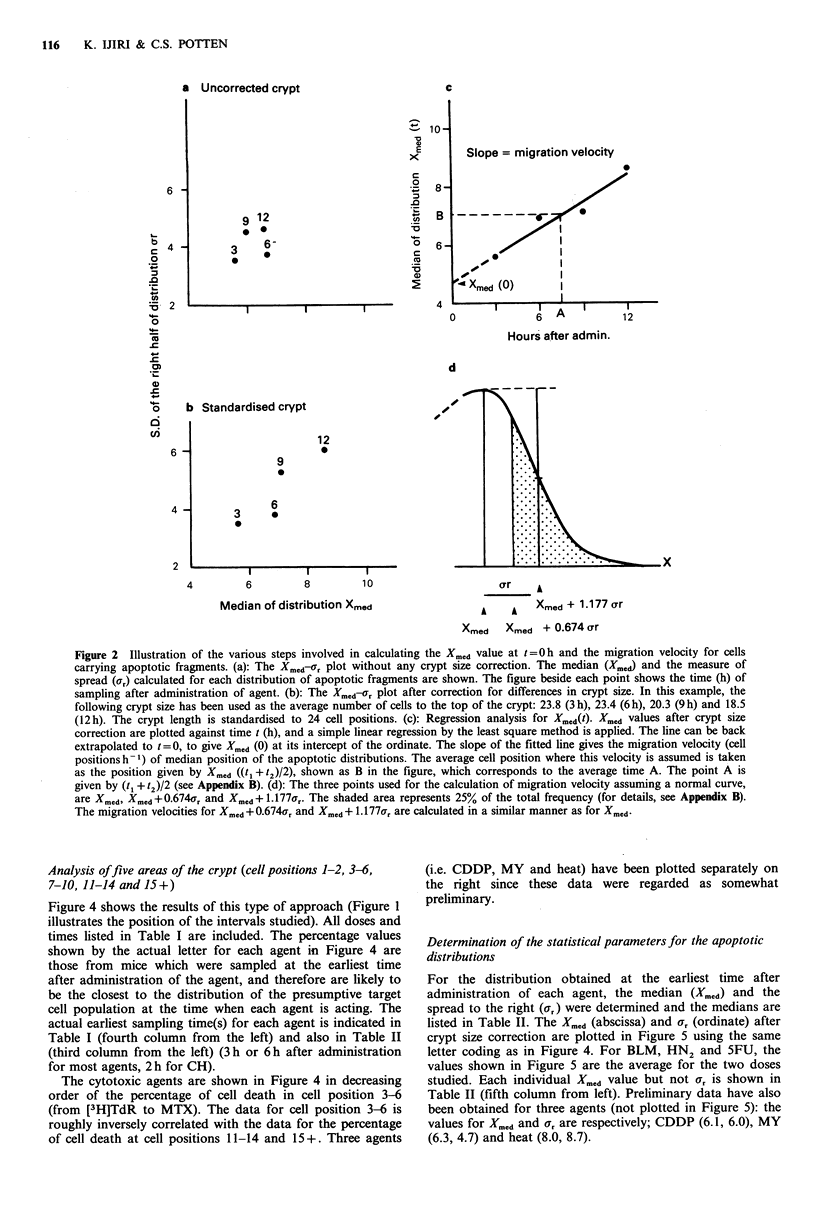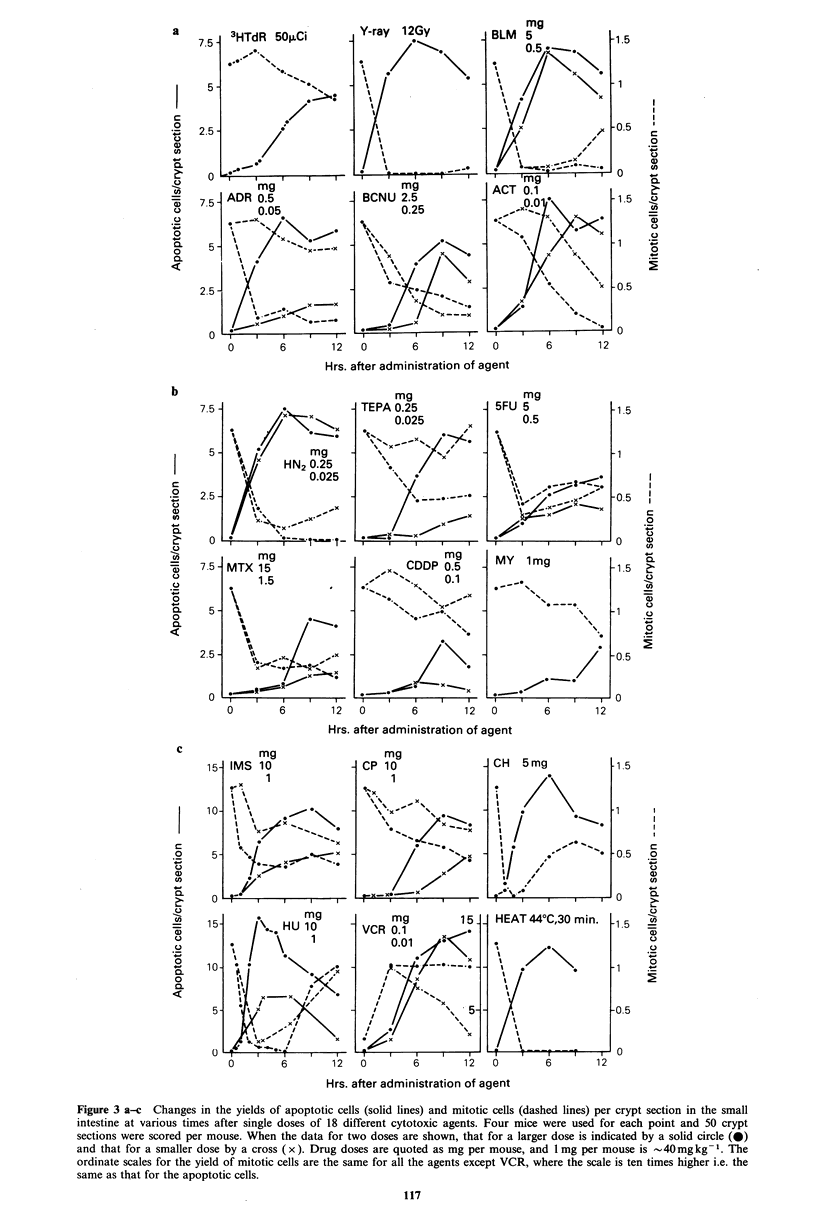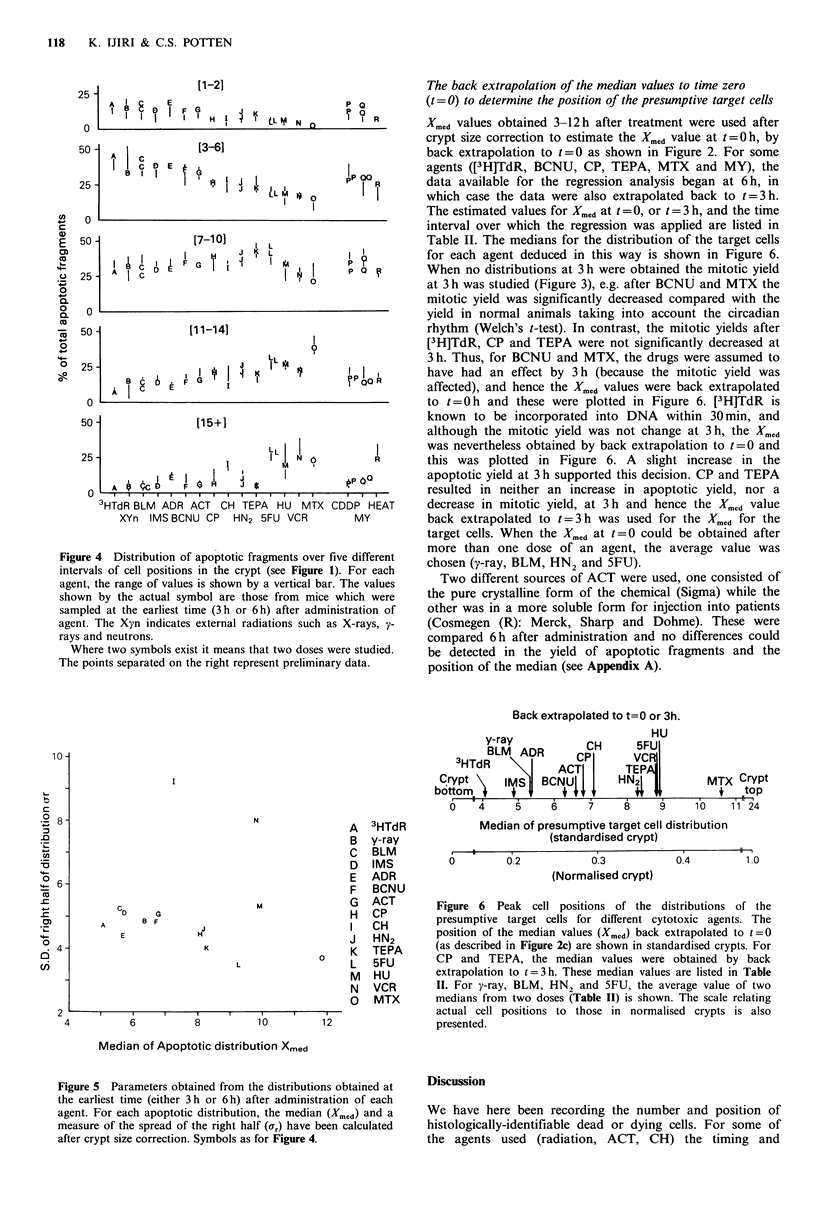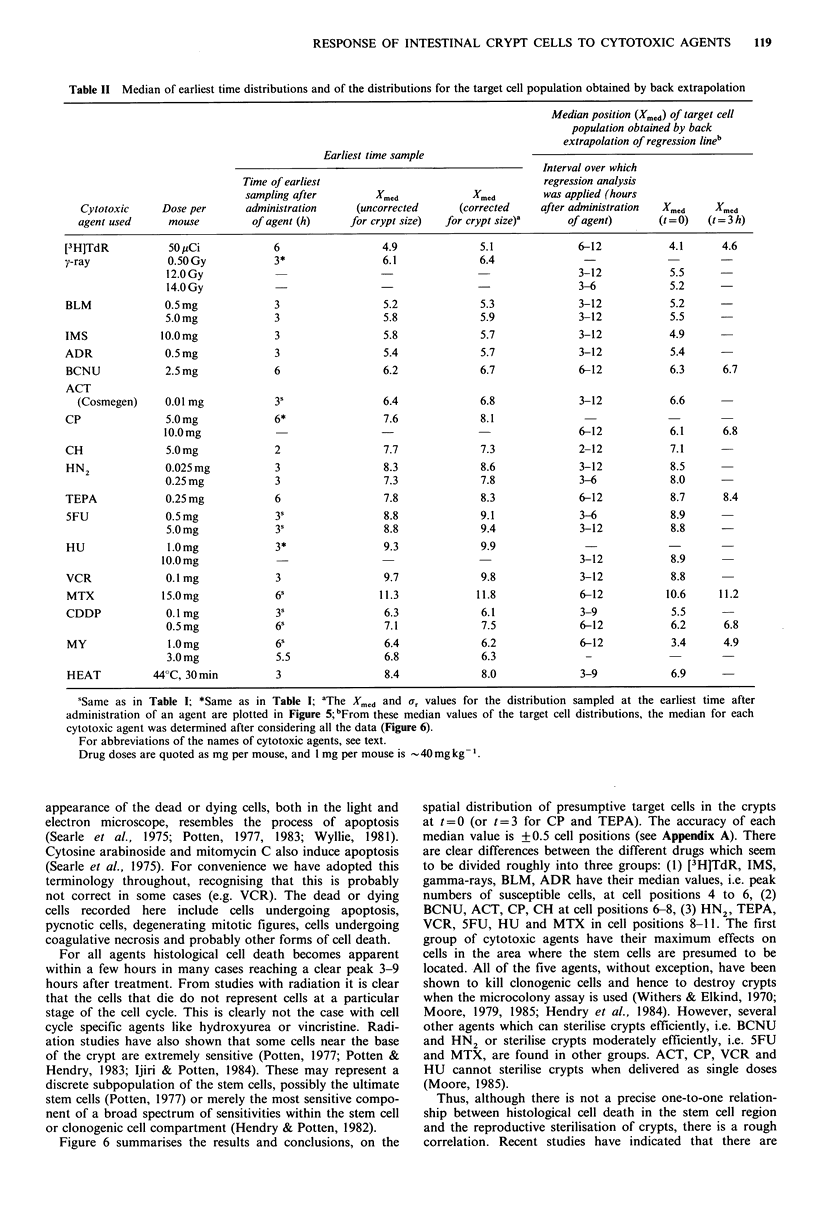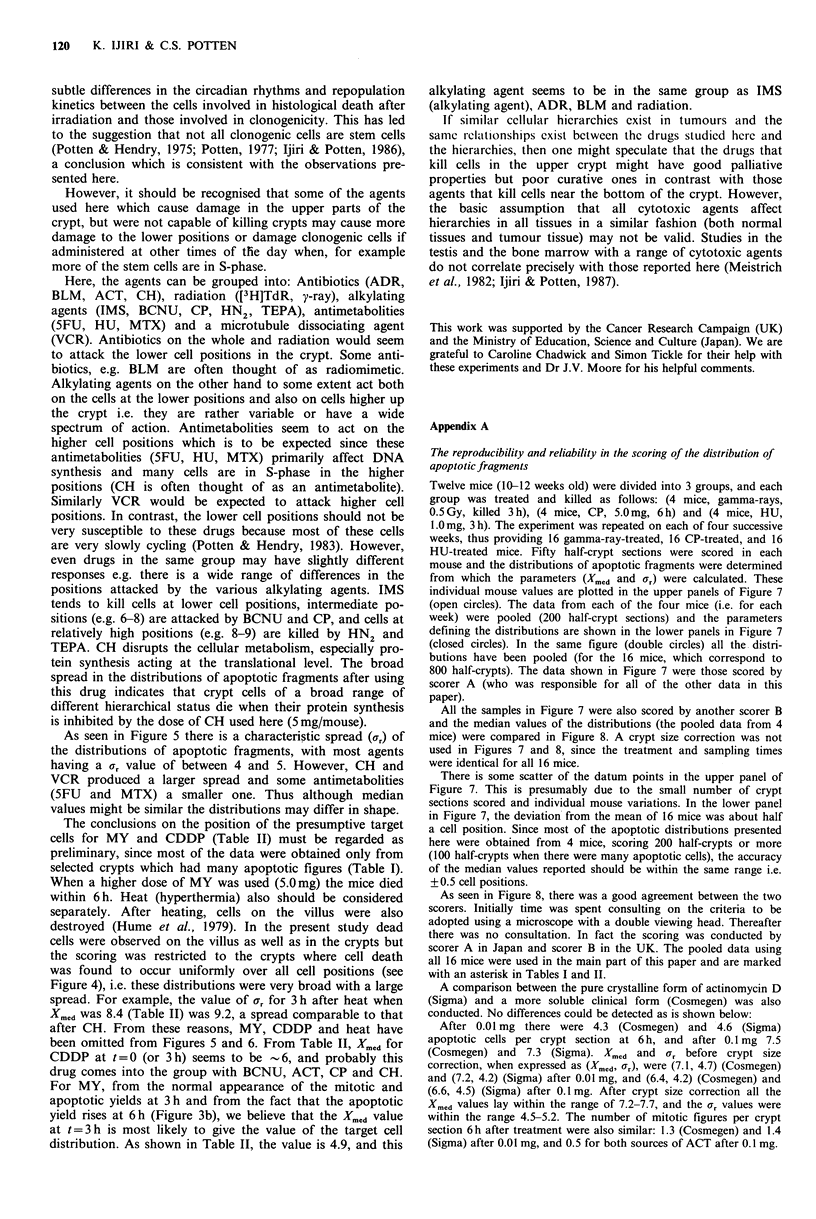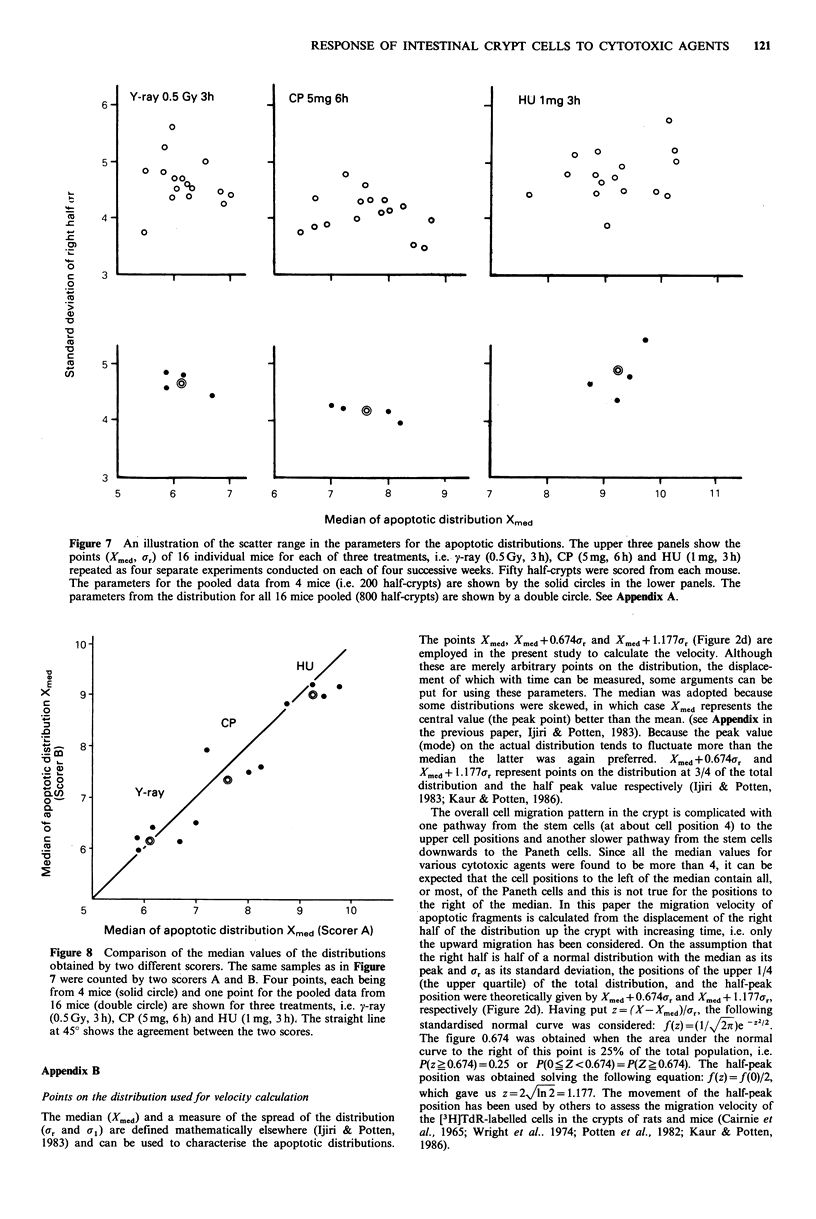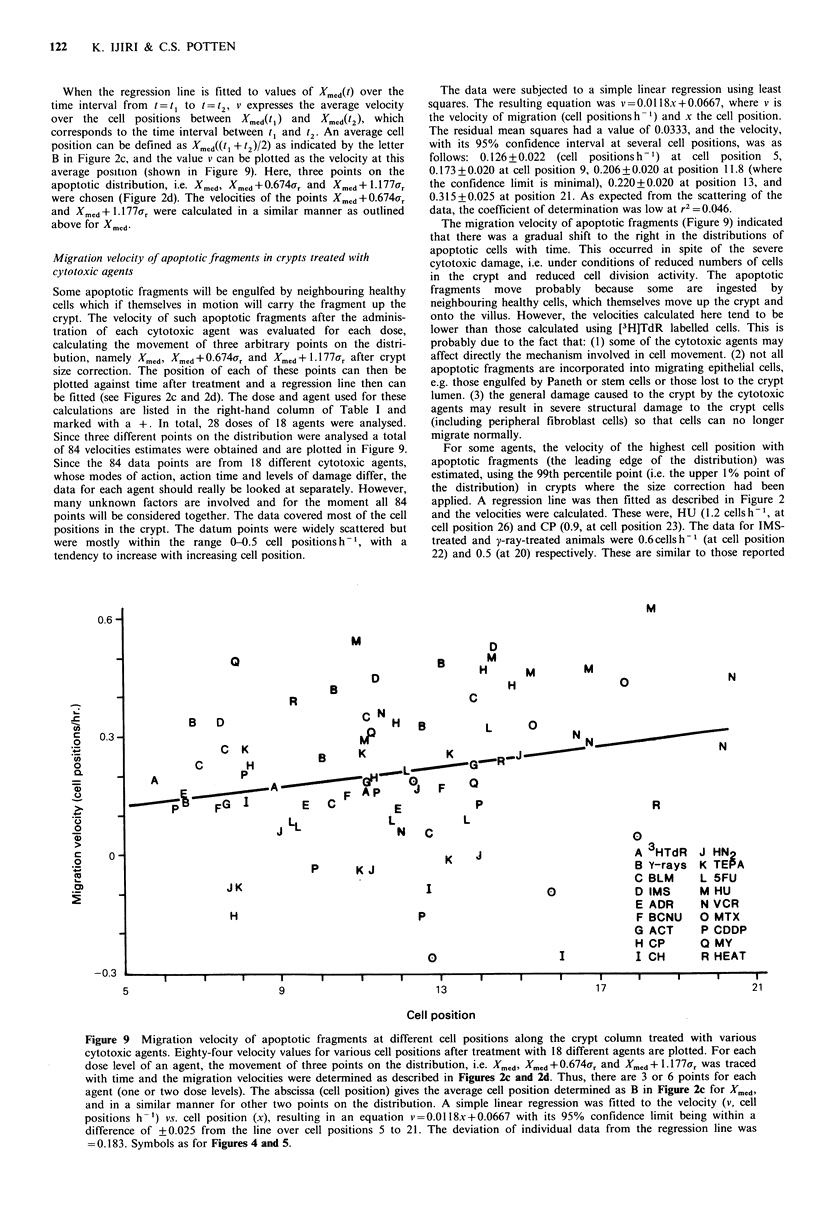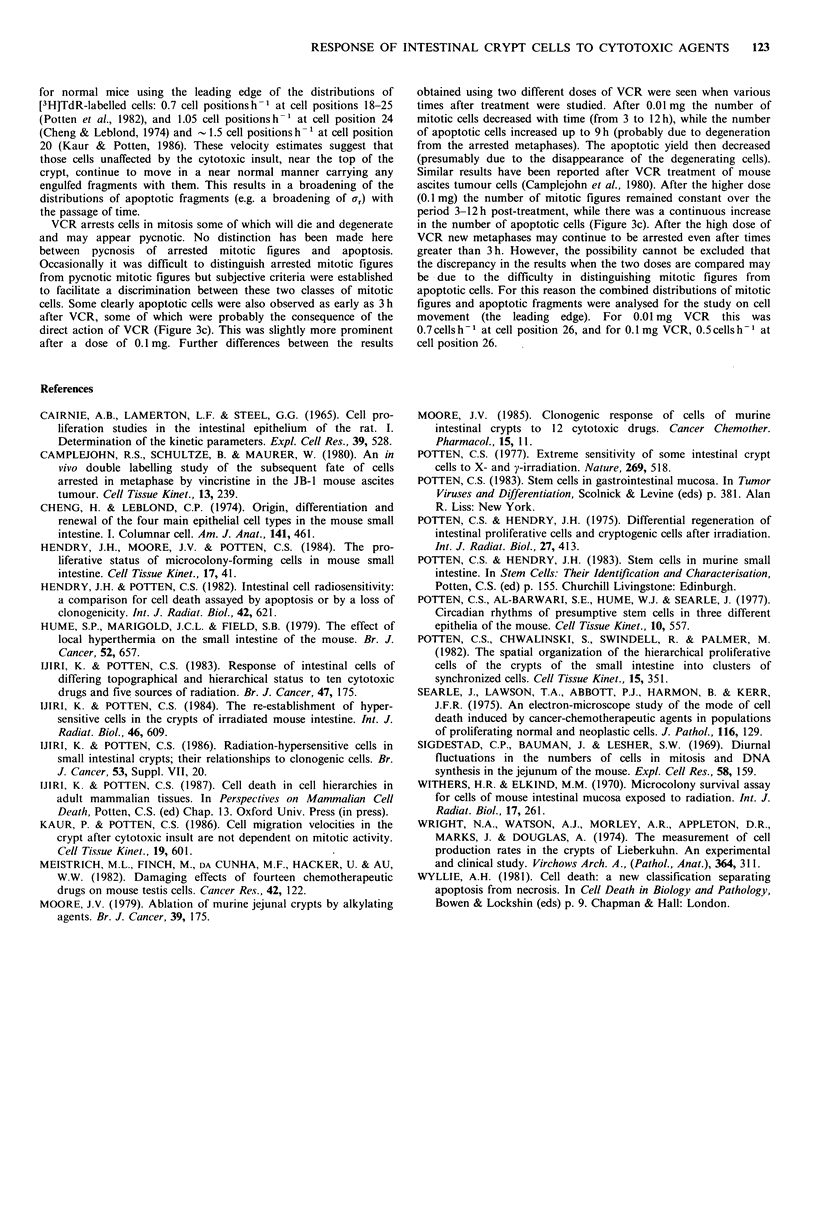# Further studies on the response of intestinal crypt cells of different hierarchical status to eighteen different cytotoxic agents.

**DOI:** 10.1038/bjc.1987.25

**Published:** 1987-02

**Authors:** K. Ijiri, C. S. Potten

## Abstract

Adult male mice were treated with one or two different doses of each of 18 different cytotoxic agents. They were sampled at various times (3-12h) thereafter, and the spatial distributions of cell death in the small intestinal crypts were studied. Dead or dying cells or cells carrying dead cell fragments were examined histologically, and all of these were recorded (for convenience as apoptotic fragments), relative to the cell position in the crypt. Thus, distributions of apoptotic fragments against cell position were determined. A regression analysis of the data obtained at different times after administration of each agent was undertaken and the position of the median of the spatial distribution of presumptive target cells was deduced for each cytotoxic agent. The accuracy of this median value was determined to be +/- 0.5 cell positions. From these median values, the different cytotoxic agents could be divided roughly into three groups: [3H]thymidine, isopropyl-methane-sulphonate, gamma-rays, bleomycin and adriamycin all have their median values (susceptible cells) at cell positions 4 to 6; bischlorethylnitrosourea, actinomycin D, cyclophosphamide and cycloheximide at cell positions 6-8; mechlorethamine, triethylenethiophosphoramide, vincristine, 5-fluorouracil, hydroxyurea and methotrexate at cell positions 8-11. The position of these medians was considered in relation to the killing of clonogenic cells. Preliminary studies on the distributions of dead cells after myleran, cis-platinum and heat (hyperthermia) were also reported. There is a general tendency for antibiotics and radiation to attack the lower cell positions in the crypt. Alkylating agents on the other hand have a somewhat broad spectrum of action. Antimetabolites and a microtubule dissociating agent act on higher cell positions. No difference could be detected between two different forms (sources) of actinomycin D. The changes in the yields of apoptotic and mitotic cells with time and the migration velocities of cells in the crypts carrying apoptotic fragments after exposure to cytotoxics are also presented.


*Br. J. Cancer* (1987), **55**, 113–123 © The Macmillan Press Ltd., 1987

### Further studies on the response of intestinal crypt cells of different hierarchical status to eighteen different cytotoxic agents

K. Ijiri[1] & C.S. Potten[2]

[1]*Zoological Institute, Faculty of Science, University of Tokyo, Hongo, Tokyo 113, Japan and* [2]*Paterson Institute for Cancer Research, Christie Hospital & Holt Radium Institute, Manchester, M20 9BX, UK.*

**Summary** Adult male mice were treated with one or two different doses of each of 18 different cytotoxic agents. They were sampled at various times (3–12 h) thereafter, and the spatial distributions of cell death in the small intestinal crypts were studied. Dead or dying cells or cells carrying dead cell fragments were examined histologically, and all of these were recorded (for convenience as apoptotic fragments), relative to the cell position in the crypt. Thus, distributions of apoptotic fragments against cell position were determined. A regression analysis of the data obtained at different times after administration of each agent was undertaken and the position of the median of the spatial distribution of presumptive target cells was deduced for each cytotoxic agent. The accuracy of this median value was determined to be $\pm 0.5$ cell positions. From these median values, the different cytotoxic agents could be divided roughly into three groups: [$^3$H]-thymidine, isopropyl-methane-sulphonate, gamma-rays, bleomycin and adriamycin all have their median values (susceptible cells) at cell positions 4 to 6; bischlorethylnitrosourea, actinomycin D, cyclophosphamide and cycloheximide at cell positions 6–8; mechlorethamine, triethylenethiophosphoramide, vincristine, 5-fluorouracil, hydroxyurea and methotrexate at cell positions 8–11. The position of these medians was considered in relation to the killing of clonogenic cells. Preliminary studies on the distributions of dead cells after myleran, *cis*-platinum and heat (hyperthermia) were also reported.

There is a general tendency for antibiotics and radiation to attack the lower cell positions in the crypt. Alkylating agents on the other hand have a somewhat broad spectrum of action. Antimetabolites and a microtubule dissociating agent act on higher cell positions. No difference could be detected between two different forms (sources) of actinomycin D. The changes in the yields of apoptotic and mitotic cells with time and the migration velocities of cells in the crypts carrying apoptotic fragments after exposure to cytotoxics are also presented.

In a previous paper (Ijiri & Potten, 1983) the response of the small intestinal crypt of the mouse to various cytotoxic agents was analysed and a heterogeneous cellular composition in the crypt was indicated. The results clearly showed that each cytotoxic agent tended to act preferentially upon a characteristically positioned band of cells up the side of the crypt. Since cell position in the crypt is likely to be related to the hierarchical status within a cell lineage, each agent appeared to have some selectivity for cells of a particular hierarchical status. The cytotoxic impact of a given agent was determined by counting the number of dead or dying cells observed histologically (pycnotic or apoptotic cells). However, in that paper the data for some of the drugs were obtained using subtly different criteria. Detailed information on the changes with time in the number of dead cells and mitotic cells was not presented. This prevented a full analysis of the regression lines through the data obtained at different times after treatment and hence we could deduce neither the cell migration rates, nor the position of the presumptive target cells for each cytotoxic agent.

Here, we extend the earlier data to cover 18 different cytotoxic agents with a full time course analysis on each.

### Materials and methods

### *Animals*

Male B6D2F1 mice ($\sim$25 g) were used when 10–12 weeks old. The animals were kept under a 12 h dark (19.00–07.00), 12 h light regimen, and were given food and water *ad libitum*. Four animals were used per experimental point.

### *Administration of drugs, radiation and heat*

All drug solutions were made up immediately before use, and dilutions were carried out either in 0.9% saline or in sterile water (except for myleran and *cis*-platinum, which were dissolved in arachis oil). All injections (0.1–0.2 ml) were given i.p. at 09.00 h. Drug does injected in this volume are quoted as mg per mouse (1 mg per mouse is $\sim$40 mg kg$^{-1}$). Whole-body irradiation was achieved using a $^{137}$Cs-gamma-irradiator at a dose rate of 4.5 Gy min$^{-1}$. Irradiation was performed between 09.00–09.30 h. Hyperthermia (44°C, 30 min) was achieved at 09.00–09.30 h by hot-bath immersion of an exteriorized loop of small intestine, as described by Hume *et al.* (1979).

### *Abbreviations and drug source*

The treatments were as follows: [$^3$H]TdR (tritiated thymidine, Amersham Int.), BLM (bleomycin hydrochloride, Nihon Kayaku, Tokyo), IMS (isopropyl-methane-sulphonate, Koch-Light), ADR (adriamycin, Montedison), BCNU (bischlorethylnitrosourea, Bristol-Myers Pharmaceuticals), ACT (actinomycin-D, purchased from Merck, Sharp and Dohme, as Cosmegen (R) or from Sigma), CP (cyclophosphamide, WB Pharmaceuticals), CH (cycloheximide, Sigma), $HN_2$ (mechlorethamine, nitrogen mustard, Boots), TEPA (triethylene-thiophosphoramide, thio-TEPA, Lederle), 5FU (5-fluorouracil, Roche), HU (hydroxyurea, Sigma), VCR (vincristine, Lilly), MTX (methotrexate, Lederle), CDDP (*cis*-diamminedichloroplatinum II; *cis*-platinum, donated by Professor B.W. Fox), MY (myleran (R), busulphan, Burroughs-Wellcome), heat (hyperthermia; 44°C, 30 min). For further details of some of the treatments see Ijiri and Potten (1983).

For some agents ([$^3$H]TdR, gamma-rays, IMS, CP, CH, HU, VCR and MY), the distributions of apoptotic fragments reported earlier (Ijiri & Potten, 1983) were reanalysed. For some drugs (ADR, ACT, $HN_2$ and 5FU) new experiments have been conducted using identical procedures which in effect repeat the earlier experiments which were obtained by courtesy of Dr J.V. Moore. Several new

Correspondence: K. Ijiri.
Received 25 June 1986; and in revised form, 6 October 1986.

drugs (BLM, BCNU, TEPA, MTX and CDDP) and heat have now been analysed and are included here. For three agents, gamma-rays, CP and HU, replicate experiments and replicate scoring were performed to define the reproducibility and reliability of the techniques (see **Appendix A**). The changes in the yield of apoptotic cells and mitotic cells with time are presented for all the 18 cytotoxic agents.

*Sample preparation, scoring of apoptotic cells and the determination of the distribution of apoptotic fragments*

Good longitudinal sections of crypts were selected i.e. crypts showing some evidence of a lumen, some Paneth cells at the base and at least 17 cells along the side. Starting at the base of the crypt column the cells were numbered up each side and the cell positions which included any apoptotic fragments were recorded. From these data a distribution of the apoptotic yield against cell position was obtained. For each experimental group, 100 to 400 half-crypts were scored, and each distribution consisted of 140–900 apoptotic fragments. The data on the reproducibility of apoptotic fragments are shown in **Appendix A**. If one or more apoptotic fragments were, from their size and clustering, thought to represent the remains of a single cell, they were recorded as a single apoptotic cell. This is clearly a subjective decision. For further details see Ijiri & Potten (1983, 1984).

*Methods of analysis for the distribution of apoptotic fragments*

A typical example of an apoptotic frequency distribution is shown in Figure 1. Actual distributions for several agents are presented elsewhere (Ijiri & Potten, 1983). The number of apoptotic fragments was recorded relative to the cell position in the crypt (Figure 1). The agents tested, their doses, and the sampling times are listed in Table I. As a general rule, distributions were only analysed if the total average yield exceeded 1.5 apoptotic cells per crypt section, i.e. were about 10 times the control level. In this way, cell death caused by the cytotoxic agent was clearly distinguished from the spontaneous level of cell death.

At some time points for several agents (ACT, TEPA, 5FU, MTX, CDDP and MY) the total average yield did not exceed 1.5 apoptotic cells per crypt section. These sections were then scored for the distribution of apoptotic fragments using selected crypts which contained many apoptoses. Such data are indicated with the superscript 's' in Table I. They were analysed in the same way as the rest of the data. In some cases where previous experiments had already accumulated sufficient data new samples were not obtained, e.g. for 9 or 12 h after the administration of some agents.

**Figure 1** Schematic illustration of the two methods of analysing the distributions of apoptotic fragments. One is to express the percentage of the total apoptotic fragments observed in various positional intervals of the crypt; five intervals in all; cell positions 1–2, 3–6, 7–10, 11–14 and 15 and beyond (15+). The other is to use statistical parameters ($X_{med}, \sigma_r, \sigma_l$) which have been defined mathematically elsewhere (Ijiri & Potten, 1983).

When two different doses were used a complete time course (3–12 h) was obtained for one at least of the doses.

The two methods of analysis for the apoptotic distributions are illustrated in Figure 1. One was to express the percentage of the total apoptotic fragments in various positional intervals of the crypt. Five intervals were generally used. The other involved statistical parameters ($X_{med}$, $\sigma_r$, $\sigma_l$) which were defined mathematically elsewhere (Ijiri & Potten, 1983). Briefly $X_{med}$ is the median cell position and $\sigma_r$ a measure of the spread of the distribution to the right and $\sigma_l$ to the left. The second method was particularly useful since it treated the crypt cell positions as continuous values, which can be treated mathematically: crypt size correction can be applied easily and it also permits the back extrapolation of the parameters to the estimated values at the time the agents were given i.e. $t=0$ h. The first method however, visually illustrates more clearly how each area in the crypt responds to the cytotoxic agents, but it is more difficult to perform the mathematical treatments when this method was used.

*Back extrapolation of the median values of the apoptotic distributions obtained at different times after treatment to estimate the target cell population*

Figure 2 illustrates this approach. In the first instance, the two parameters, the median ($X_{med}$) and $\sigma_r$ were calculated for each distribution of apoptotic fragments at each time after the administration of an agent (see Figure 1). Figure 2a shows a typical example of the relationship between $X_{med}$ and $\sigma_r$ for the 3–12 h data for one dose level of an agent. In this example, at 6 h, both $X_{med}$ and $\sigma_r$ have larger values than at 3 h, which indicates a shift of the apoptotic distribution to the right (i.e. towards the crypt top). The increase in $\sigma_r$ means that cells in the upper part of the crypt move more rapidly than those in the lower part, causing the tail on the distribution to increase with time. In some cases, the 9 h data have a lower value of $X_{med}$ and a larger $\sigma_r$ compared with the 6 h data. Similar changes may be noted for the 12 h data. This is due to the fact that cell death and cell loss have shortened the crypt. After applying a correction for crypt size (described below), the pairs of coordinates ($X_{med}$, $\sigma_r$) in Figure 2a appear as in Figure 2b. Both parameters now increase steadily with increasing time.

When $X_{med}$ values obtained after crypt size correction are plotted against time (Figure 2c), a least square linear regression line can be fitted for the equation $X_{med}(t)=vt+X_{med}(0)$, the median can be back extrapolated to $t=0$ ($X_{med}(0)$). The migration velocity $v$ (expressed in cell positions $h^{-1}$) can also be extracted as the slope of the line (see **Appendix B**).

*Correction for crypt size*

A crypt size correction was achieved by multiplying each value ($X_{med}$ or $\sigma_r$) by a factor (F) given by C/T where C = average number of cells to the top of the crypt in untreated control animals and T = the average number to the top of the crypt in treated animals. C was taken as 24 cells, the value used in the previous paper (Ijiri & Potten, 1983), i.e. the crypt length was standardised to 24 cell positions, which was also the approximate value for untreated animals in the present experiments.

### Results

*Apoptotic and mitotic figures*

Figure 3 shows the results for the number of apoptotic cells and mitotic figures expressed per crypt section at various times (3–12 h) after the administration of single doses of 18 different cytotoxic agents. It has been reported that the mitotic yield in small intestinal crypts exhibits a circadian rhythm (Sigdestad *et al.*, 1969, Potten *et al.*, 1977). A *t*-test

**Table I** Cytotoxic agents, range of doses and post-treatment sampling times and kinds of analysis for apoptotic distributions

| *Cytotoxic agent used* | *Dose per mouse* | *Time of sampling after administration (h)* | *Time of earliest sampling (h) after administration of agent*[a] | *Regression analysis for target cell position*[b] | *Regression analysis for migration velocity*[c] |
|---|---|---|---|---|---|
| [$^3$H]TdR | 50 μCi | 6, 6.5, 9, 12 | 6 | + | + |
| External- | 0.50 Gy | 3* | 3* | | |
| radiations | 12.0 Gy | 3, 6, 9, 12 | | + | + |
| γ-ray | 14.0 Gy | 3, 6 | | + | + |
| Two experiments using γ-rays, one using X-rays, two using neutrons were also analysed (for details, see Ijiri & Potten, 1983) | | | | | |
| BLM | 0.5 mg | 3, 6, 9, 12 | 3 | + | + |
| | 5.0 mg | 3, 6, 9, 12 | 3 | + | + |
| IMS | 10.0 mg | 3, 6, 9, 12 | 3 | + | + |
| ADR | 0.5 mg | 3, 6, 9, 12 | 3 | + | + |
| BCNU | 0.25 mg | 9 | | | |
| | 2.5 mg | 6, 9, 12 | 6 | + | + |
| ACT | | | | | |
| (Cosmegen) | 0.01 mg | $3^s$, 6, 9, 12 | $3^s$ | + | + |
| | 0.1 mg | 6 | | | |
| (Sigma) | 0.01 mg | 6 | | | |
| | 0.1 mg | 6 | | | |
| CP | 1.0 mg | 9, 12 | | | + |
| | 5.0 mg | 6* | 6* | | |
| | 10.0 mg | 6, 9, 12 | | + | + |
| CH | 5.0 mg | 2, 3, 6, 12 | 2 | + | + |
| $HN_2$ | 0.025 mg | 3, 6, 9, 12 | 3 | + | + |
| | 0.25 mg | 3, 6 | 3 | + | + |
| TEPA | 0.025 mg | $9^s$, $12^s$ | | | + |
| | 0.25 mg | 6, 9, 12 | 6 | + | + |
| 5FU | 0.5 mg | $3^s$, 6 | $3^s$ | + | + |
| | 5.0 mg | $3^s$, 6, 9, 12 | $3^s$ | + | + |
| HU | 1.0 mg | 3* | 3* | | |
| | 1.0 mg | 3, 3.5, 6.5 | | | + |
| | 10.0 mg | 3, 4, 5, 6, 9, 12 | | + | + |
| VCR | 0.01 mg | 6, 9, 12 | | | + |
| | 0.1 mg | 3, 6, 9, 12 | 3 | + | + |
| MTX | 1.5 mg | $9^s$, $12^s$ | | | + |
| | 15.0 mg | $6^s$, 9, 12 | $6^s$ | + | + |
| CDDP | 0.1 mg | $3^s$, $6^s$, $9^s$ | $3^s$ | + | + |
| | 0.5 mg | $6^s$, 9, $12^s$ | $6^s$ | + | + |
| MY | 1.0 mg | $6^s$, $9^s$, 12 | $6^s$ | + | + |
| | 3.0 mg | 5.5 | 5.5 | | |
| HEAT | 44°C, 30 min | 3, 6, 9 | 3 | + | + |

[s]Data obtained by scoring selected crypts which contained many apoptotic cells: a total average yield exceeding 1.5 apoptotic cells per crypt section; *The sampling time for pooled data using 16 mice (800 half-crypts), see **Appendix A**; [a]The earliest time after administration of agent when the distribution of apoptotic fragments was high enough to distinguish it from background (see text). These values have been used in **Figures 4 and 5** and **Table II**; [b]When a regression analysis was applied (indicated by a +) the parameters for the target cell population were obtained by back extrapolation to $t=0$ or 3 h. Such values are shown in **Table II** and **Figure 6**; [c]When a regression analysis was applied (indicated by a +) the migration velocity of the cells carrying apoptotic fragments can be calculated. The velocities for three points on the distribution were estimated and are plotted in **Figure 9**.

For abbreviations of the names of cytotoxic agents, see text.

Drug doses are quoted as mg per mouse, and 1 mg per mouse is $\sim$40 mg kg$^{-1}$.

was applied to detect the time when the mitotic yield in the treated mice was significantly below the yield in normal animals at each time of the day. From the results of the statistical test, the decrease in mitotic yield (dotted line in Figure 3) generally coincided with, or preceded, the rise in apoptotic cells (solid line). Five agents were exceptions to this rule. For four of them ([$^3$H]TdR, ACT, CDDP and MY), the decrease in the mitotic yield to values significantly lower than that in normal animals followed the rise in apoptotic yield. The other agent was VCR. This is a stathmokinetic agent, arresting cells at metaphase. Most of the arrested cells will eventually die. They do so in mitosis and so differ from the other agents where death may occur from other phases of the cell cycle. For convenience, cell death induced by VCR has been loosely given the same term (apoptosis). At early times after VCR administration, apoptotic yield and mitotic yield rose in parallel. The results after VCR are also presented in Figure 3c. In Figure 3, drug doses are quoted as mg per mouse, and 1 mg per mouse is $\sim$40 mg kg$^{-1}$.

**Figure 2** Illustration of the various steps involved in calculating the $X_{med}$ value at $t=0$ h and the migration velocity for cells carrying apoptotic fragments. (a): The $X_{med}$–$\sigma_r$ plot without any crypt size correction. The median ($X_{med}$) and the measure of spread ($\sigma_r$) calculated for each distribution of apoptotic fragments are shown. The figure beside each point shows the time (h) of sampling after administration of agent. (b): The $X_{med}$–$\sigma_r$ plot after correction for differences in crypt size. In this example, the following crypt size has been used as the average number of cells to the top of the crypt: 23.8 (3 h), 23.4 (6 h), 20.3 (9 h) and 18.5 (12 h). The crypt length is standardised to 24 cell positions. (c): Regression analysis for $X_{med}(t)$. $X_{med}$ values after crypt size correction are plotted against time $t$ (h), and a simple linear regression by the least square method is applied. The line can be back extrapolated to $t=0$, to give $X_{med}$ (0) at its intercept of the ordinate. The slope of the fitted line gives the migration velocity (cell positions $h^{-1}$) of median position of the apoptotic distributions. The average cell position where this velocity is assumed is taken as the position given by $X_{med}$ $((t_1+t_2)/2)$, shown as B in the figure, which corresponds to the average time A. The point A is given by $(t_1+t_2)/2$ (see **Appendix B**). (d): The three points used for the calculation of migration velocity assuming a normal curve, are $X_{med}$, $X_{med}+0.674\sigma_r$ and $X_{med}+1.177\sigma_r$. The shaded area represents 25% of the total frequency (for details, see **Appendix B**). The migration velocities for $X_{med}+0.674\sigma_r$ and $X_{med}+1.177\sigma_r$ are calculated in a similar manner as for $X_{med}$.

*Analysis of five areas of the crypt (cell positions 1–2, 3–6, 7–10, 11–14 and 15+)*

Figure 4 shows the results of this type of approach (Figure 1 illustrates the position of the intervals studied). All doses and times listed in Table I are included. The percentage values shown by the actual letter for each agent in Figure 4 are those from mice which were sampled at the earliest time after administration of the agent, and therefore are likely to be the closest to the distribution of the presumptive target cell population at the time when each agent is acting. The actual earliest sampling time(s) for each agent is indicated in Table I (fourth column from the left) and also in Table II (third column from the left) (3 h or 6 h after administration for most agents, 2 h for CH).

The cytotoxic agents are shown in Figure 4 in decreasing order of the percentage of cell death in cell position 3–6 (from [$^3$H]TdR to MTX). The data for cell position 3–6 is roughly inversely correlated with the data for the percentage of cell death at cell positions 11–14 and 15+. Three agents (i.e. CDDP, MY and heat) have been plotted separately on the right since these data were regarded as somewhat preliminary.

*Determination of the statistical parameters for the apoptotic distributions*

For the distribution obtained at the earliest time after administration of each agent, the median ($X_{med}$) and the spread to the right ($\sigma_r$) were determined and the medians are listed in Table II. The $X_{med}$ (abscissa) and $\sigma_r$ (ordinate) after crypt size correction are plotted in Figure 5 using the same letter coding as in Figure 4. For BLM, $HN_2$ and 5FU, the values shown in Figure 5 are the average for the two doses studied. Each individual $X_{med}$ value but not $\sigma_r$ is shown in Table II (fifth column from left). Preliminary data have also been obtained for three agents (not plotted in Figure 5): the values for $X_{med}$ and $\sigma_r$ are respectively; CDDP (6.1, 6.0), MY (6.3, 4.7) and heat (8.0, 8.7).

**Figure 3 a–c** Changes in the yields of apoptotic cells (solid lines) and mitotic cells (dashed lines) per crypt section in the small intestine at various times after single doses of 18 different cytotoxic agents. Four mice were used for each point and 50 crypt sections were scored per mouse. When the data for two doses are shown, that for a larger dose is indicated by a solid circle (●) and that for a smaller dose by a cross (×). Drug doses are quoted as mg per mouse, and 1 mg per mouse is ~40 mg kg⁻¹. The ordinate scales for the yield of mitotic cells are the same for all the agents except VCR, where the scale is ten times higher i.e. the same as that for the apoptotic cells.

**Figure 4** Distribution of apoptotic fragments over five different intervals of cell positions in the crypt (see **Figure 1**). For each agent, the range of values is shown by a vertical bar. The values shown by the actual symbol are those from mice which were sampled at the earliest time (3 h or 6 h) after administration of agent. The X$\gamma$n indicates external radiations such as X-rays, $\gamma$-rays and neutrons.

Where two symbols exist it means that two doses were studied. The points separated on the right represent preliminary data.

**Figure 5** Parameters obtained from the distributions obtained at the earliest time (either 3 h or 6 h) after administration of each agent. For each apoptotic distribution, the median ($X_{med}$) and a measure of the spread of the right half ($\sigma_r$) have been calculated after crypt size correction. Symbols as for **Figure 4**.

*The back extrapolation of the median values to time zero ($t=0$) to determine the position of the presumptive target cells*

$X_{med}$ values obtained 3–12 h after treatment were used after crypt size correction to estimate the $X_{med}$ value at $t=0$ h, by back extrapolation to $t=0$ as shown in Figure 2. For some agents ([$^3$H]TdR, BCNU, CP, TEPA, MTX and MY), the data available for the regression analysis began at 6 h, in which case the data were also extrapolated back to $t=3$ h. The estimated values for $X_{med}$ at $t=0$, or $t=3$ h, and the time interval over which the regression was applied are listed in Table II. The medians for the distribution of the target cells for each agent deduced in this way is shown in Figure 6. When no distributions at 3 h were obtained the mitotic yield at 3 h was studied (Figure 3), e.g. after BCNU and MTX the mitotic yield was significantly decreased compared with the yield in normal animals taking into account the circadian rhythm (Welch's $t$-test). In contrast, the mitotic yields after [$^3$H]TdR, CP and TEPA were not significantly decreased at 3 h. Thus, for BCNU and MTX, the drugs were assumed to have had an effect by 3 h (because the mitotic yield was affected), and hence the $X_{med}$ values were back extrapolated to $t=0$ h and these were plotted in Figure 6. [$^3$H]TdR is known to be incorporated into DNA within 30 min, and although the mitotic yield was not change at 3 h, the $X_{med}$ was nevertheless obtained by back extrapolation to $t=0$ and this was plotted in Figure 6. A slight increase in the apoptotic yield at 3 h supported this decision. CP and TEPA resulted in neither an increase in apoptotic yield, nor a decrease in mitotic yield, at 3 h and hence the $X_{med}$ value back extrapolated to $t=3$ h was used for the $X_{med}$ for the target cells. When the $X_{med}$ at $t=0$ could be obtained after more than one dose of an agent, the average value was chosen ($\gamma$-ray, BLM, $HN_2$ and 5FU).

Two different sources of ACT were used, one consisted of the pure crystalline form of the chemical (Sigma) while the other was in a more soluble form for injection into patients (Cosmegen (R): Merck, Sharp and Dohme). These were compared 6 h after administration and no differences could be detected in the yield of apoptotic fragments and the position of the median (see **Appendix A**).

**Figure 6** Peak cell positions of the distributions of the presumptive target cells for different cytotoxic agents. The position of the median values ($X_{med}$) back extrapolated to $t=0$ (as described in **Figure 2c**) are shown in standardised crypts. For CP and TEPA, the median values were obtained by back extrapolation to $t=3$ h. These median values are listed in **Table II.** For $\gamma$-ray, BLM, $HN_2$ and 5FU, the average value of two medians from two doses (**Table II**) is shown. The scale relating actual cell positions to those in normalised crypts is also presented.

### Discussion

We have here been recording the number and position of histologically-identifiable dead or dying cells. For some of the agents used (radiation, ACT, CH) the timing and

**Table II** Median of earliest time distributions and of the distributions for the target cell population obtained by back extrapolation

| | | *Earliest time sample* | | | *Median position ($X_{med}$) of target cell population obtained by back extrapolation of regression line*[b] | | |
|---|---|---|---|---|---|---|---|
| *Cytotoxic agent used* | *Dose per mouse* | *Time of earliest sampling after administration of agent (h)* | *$X_{med}$ (uncorrected for crypt size)* | *$X_{med}$ (corrected for crypt size)*[a] | *Interval over which regression analysis was applied (hours after administration of agent)* | *$X_{med}$ ($t=0$)* | *$X_{med}$ ($t=3h$)* |
| [$^3$H]TdR | 50 μCi | 6 | 4.9 | 5.1 | 6–12 | 4.1 | 4.6 |
| γ-ray | 0.50 Gy | 3* | 6.1 | 6.4 | — | — | — |
| | 12.0 Gy | — | — | — | 3–12 | 5.5 | — |
| | 14.0 Gy | — | — | — | 3–6 | 5.2 | — |
| BLM | 0.5 mg | 3 | 5.2 | 5.3 | 3–12 | 5.2 | — |
| | 5.0 mg | 3 | 5.8 | 5.9 | 3–12 | 5.5 | — |
| IMS | 10.0 mg | 3 | 5.8 | 5.7 | 3–12 | 4.9 | — |
| ADR | 0.5 mg | 3 | 5.4 | 5.7 | 3–12 | 5.4 | — |
| BCNU | 2.5 mg | 6 | 6.2 | 6.7 | 6–12 | 6.3 | 6.7 |
| ACT (Cosmegen) | 0.01 mg | 3[s] | 6.4 | 6.8 | 3–12 | 6.6 | — |
| CP | 5.0 mg | 6* | 7.6 | 8.1 | — | — | — |
| | 10.0 mg | — | — | — | 6–12 | 6.1 | 6.8 |
| CH | 5.0 mg | 2 | 7.7 | 7.3 | 2–12 | 7.1 | — |
| $HN_2$ | 0.025 mg | 3 | 8.3 | 8.6 | 3–12 | 8.5 | — |
| | 0.25 mg | 3 | 7.3 | 7.8 | 3–6 | 8.0 | — |
| TEPA | 0.25 mg | 6 | 7.8 | 8.3 | 6–12 | 8.7 | 8.4 |
| 5FU | 0.5 mg | 3[s] | 8.8 | 9.1 | 3–6 | 8.9 | — |
| | 5.0 mg | 3[s] | 8.8 | 9.4 | 3–12 | 8.8 | — |
| HU | 1.0 mg | 3* | 9.3 | 9.9 | — | — | — |
| | 10.0 mg | — | — | — | 3–12 | 8.9 | — |
| VCR | 0.1 mg | 3 | 9.7 | 9.8 | 3–12 | 8.8 | — |
| MTX | 15.0 mg | 6[s] | 11.3 | 11.8 | 6–12 | 10.6 | 11.2 |
| CDDP | 0.1 mg | 3[s] | 6.3 | 6.1 | 3–9 | 5.5 | — |
| | 0.5 mg | 6[s] | 7.1 | 7.5 | 6–12 | 6.2 | 6.8 |
| MY | 1.0 mg | 6[s] | 6.4 | 6.2 | 6–12 | 3.4 | 4.9 |
| | 3.0 mg | 5.5 | 6.8 | 6.3 | - | — | — |
| HEAT | 44°C, 30 min | 3 | 8.4 | 8.0 | 3–9 | 6.9 | — |

[s]Same as in **Table I**; *Same as in **Table I**; [a]The $X_{med}$ and $\sigma_r$ values for the distribution sampled at the earliest time after administration of an agent are plotted in **Figure 5**; [b]From these median values of the target cell distributions, the median for each cytotoxic agent was determined after considering all the data (**Figure 6**).

For abbreviations of the names of cytotoxic agents, see text.

Drug doses are quoted as mg per mouse, and 1 mg per mouse is ~40 mg kg$^{-1}$.

appearance of the dead or dying cells, both in the light and electron microscope, resembles the process of apoptosis (Searle *et al.*, 1975; Potten, 1977, 1983; Wyllie, 1981). Cytosine arabinoside and mitomycin C also induce apoptosis (Searle *et al.*, 1975). For convenience we have adopted this terminology throughout, recognising that this is probably not correct in some cases (e.g. VCR). The dead or dying cells recorded here include cells undergoing apoptosis, pycnotic cells, degenerating mitotic figures, cells undergoing coagulative necrosis and probably other forms of cell death.

For all agents histological cell death becomes apparent within a few hours in many cases reaching a clear peak 3–9 hours after treatment. From studies with radiation it is clear that the cells that die do not represent cells at a particular stage of the cell cycle. This is clearly not the case with cell cycle specific agents like hydroxyurea or vincristine. Radiation studies have also shown that some cells near the base of the crypt are extremely sensitive (Potten, 1977; Potten & Hendry, 1983; Ijiri & Potten, 1984). These may represent a discrete subpopulation of the stem cells, possibly the ultimate stem cells (Potten, 1977) or merely the most sensitive component of a broad spectrum of sensitivities within the stem cell or clonogenic cell compartment (Hendry & Potten, 1982).

Figure 6 summarises the results and conclusions, on the spatial distribution of presumptive target cells in the crypts at $t=0$ (or $t=3$ for CP and TEPA). The accuracy of each median value is $\pm 0.5$ cell positions (see **Appendix** A). There are clear differences between the different drugs which seem to be divided roughly into three groups: (1) [$^3$H]TdR, IMS, gamma-rays, BLM, ADR have their median values, i.e. peak numbers of susceptible cells, at cell positions 4 to 6, (2) BCNU, ACT, CP, CH at cell positions 6–8, (3) $HN_2$, TEPA, VCR, 5FU, HU and MTX in cell positions 8–11. The first group of cytotoxic agents have their maximum effects on cells in the area where the stem cells are presumed to be located. All of the five agents, without exception, have been shown to kill clonogenic cells and hence to destroy crypts when the microcolony assay is used (Withers & Elkind, 1970; Moore, 1979, 1985; Hendry *et al.*, 1984). However, several other agents which can sterilise crypts efficiently, i.e. BCNU and $HN_2$ or sterilise crypts moderately efficiently, i.e. 5FU and MTX, are found in other groups. ACT, CP, VCR and HU cannot sterilise crypts when delivered as single doses (Moore, 1985).

Thus, although there is not a precise one-to-one relationship between histological cell death in the stem cell region and the reproductive sterilisation of crypts, there is a rough correlation. Recent studies have indicated that there are subtle differences in the circadian rhythms and repopulation kinetics between the cells involved in histological death after irradiation and those involved in clonogenicity. This has led to the suggestion that not all clonogenic cells are stem cells (Potten & Hendry, 1975; Potten, 1977; Ijiri & Potten, 1986), a conclusion which is consistent with the observations presented here.

However, it should be recognised that some of the agents used here which cause damage in the upper parts of the crypt, but were not capable of killing crypts may cause more damage to the lower positions or damage clonogenic cells if administered at other times of the day when, for example more of the stem cells are in S-phase.

Here, the agents can be grouped into: Antibiotics (ADR, BLM, ACT, CH), radiation ([$^3$H]TdR, $\gamma$-ray), alkylating agents (IMS, BCNU, CP, $HN_2$, TEPA), antimetabolities (5FU, HU, MTX) and a microtubule dissociating agent (VCR). Antibiotics on the whole and radiation would seem to attack the lower cell positions in the crypt. Some antibiotics, e.g. BLM are often thought of as radiomimetic. Alkylating agents on the other hand to some extent act both on the cells at the lower positions and also on cells higher up the crypt i.e. they are rather variable or have a wide spectrum of action. Antimetabolities seem to act on the higher cell positions which is to be expected since these antimetabolities (5FU, HU, MTX) primarily affect DNA synthesis and many cells are in S-phase in the higher positions (CH is often thought of as an antimetabolite). Similarly VCR would be expected to attack higher cell positions. In contrast, the lower cell positions should not be very susceptible to these drugs because most of these cells are very slowly cycling (Potten & Hendry, 1983). However, even drugs in the same group may have slightly different responses e.g. there is a wide range of differences in the positions attacked by the various alkylating agents. IMS tends to kill cells at lower cell positions, intermediate positions (e.g. 6–8) are attacked by BCNU and CP, and cells at relatively high positions (e.g. 8–9) are killed by $HN_2$ and TEPA. CH disrupts the cellular metabolism, especially protein synthesis acting at the translational level. The broad spread in the distributions of apoptotic fragments after using this drug indicates that crypt cells of a broad range of different hierarchical status die when their protein synthesis is inhibited by the dose of CH used here (5 mg/mouse).

As seen in Figure 5 there is a characteristic spread ($\sigma_r$) of the distributions of apoptotic fragments, with most agents having a $\sigma_r$ value of between 4 and 5. However, CH and VCR produced a larger spread and some antimetabolities (5FU and MTX) a smaller one. Thus although median values might be similar the distributions may differ in shape.

The conclusions on the position of the presumptive target cells for MY and CDDP (Table II) must be regarded as preliminary, since most of the data were obtained only from selected crypts which had many apoptotic figures (Table I). When a higher dose of MY was used (5.0 mg) the mice died within 6 h. Heat (hyperthermia) also should be considered separately. After heating, cells on the villus were also destroyed (Hume *et al.*, 1979). In the present study dead cells were observed on the villus as well as in the crypts but the scoring was restricted to the crypts where cell death was found to occur uniformly over all cell positions (see Figure 4), i.e. these distributions were very broad with a large spread. For example, the value of $\sigma_r$ for 3 h after heat when $X_{med}$ was 8.4 (Table II) was 9.2, a spread comparable to that after CH. From these reasons, MY, CDDP and heat have been omitted from Figures 5 and 6. From Table II, $X_{med}$ for CDDP at $t=0$ (or 3 h) seems to be $\sim$6, and probably this drug comes into the group with BCNU, ACT, CP and CH. For MY, from the normal appearance of the mitotic and apoptotic yields at 3 h and from the fact that the apoptotic yield rises at 6 h (Figure 3b), we believe that the $X_{med}$ value at $t=3$ h is most likely to give the value of the target cell distribution. As shown in Table II, the value is 4.9, and this alkylating agent seems to be in the same group as IMS (alkylating agent), ADR, BLM and radiation.

If similar cellular hierarchies exist in tumours and the same relationships exist between the drugs studied here and the hierarchies, then one might speculate that the drugs that kill cells in the upper crypt might have good palliative properties but poor curative ones in contrast with those agents that kill cells near the bottom of the crypt. However, the basic assumption that all cytotoxic agents affect hierarchies in all tissues in a similar fashion (both normal tissues and tumour tissue) may not be valid. Studies in the testis and the bone marrow with a range of cytotoxic agents do not correlate precisely with those reported here (Meistrich *et al.*, 1982; Ijiri & Potten, 1987).

This work was supported by the Cancer Research Campaign (UK) and the Ministry of Education, Science and Culture (Japan). We are grateful to Caroline Chadwick and Simon Tickle for their help with these experiments and Dr J.V. Moore for his helpful comments.

### Appendix A

*The reproducibility and reliability in the scoring of the distribution of apoptotic fragments*

Twelve mice (10–12 weeks old) were divided into 3 groups, and each group was treated and killed as follows: (4 mice, gamma-rays, 0.5 Gy, killed 3 h), (4 mice, CP, 5.0 mg, 6 h) and (4 mice, HU, 1.0 mg, 3 h). The experiment was repeated on each of four successive weeks, thus providing 16 gamma-ray-treated, 16 CP-treated, and 16 HU-treated mice. Fifty half-crypt sections were scored in each mouse and the distributions of apoptotic fragments were determined from which the parameters ($X_{med}$ and $\sigma_r$) were calculated. These individual mouse values are plotted in the upper panels of Figure 7 (open circles). The data from each of the four mice (i.e. for each week) were pooled (200 half-crypt sections) and the parameters defining the distributions are shown in the lower panels in Figure 7 (closed circles). In the same figure (double circles) all the distributions have been pooled (for the 16 mice, which correspond to 800 half-crypts). The data shown in Figure 7 were those scored by scorer A (who was responsible for all of the other data in this paper).

All the samples in Figure 7 were also scored by another scorer B and the median values of the distributions (the pooled data from 4 mice) were compared in Figure 8. A crypt size correction was not used in Figures 7 and 8, since the treatment and sampling times were identical for all 16 mice.

There is some scatter of the datum points in the upper panel of Figure 7. This is presumably due to the small number of crypt sections scored and individual mouse variations. In the lower panel in Figure 7, the deviation from the mean of 16 mice was about half a cell position. Since most of the apoptotic distributions presented here were obtained from 4 mice, scoring 200 half-crypts or more (100 half-crypts when there were many apoptotic cells), the accuracy of the median values reported should be within the same range i.e. $\pm 0.5$ cell positions.

As seen in Figure 8, there was a good agreement between the two scorers. Initially time was spent consulting on the criteria to be adopted using a microscope with a double viewing head. Thereafter there was no consultation. In fact the scoring was conducted by scorer A in Japan and scorer B in the UK. The pooled data using all 16 mice were used in the main part of this paper and are marked with an asterisk in Tables I and II.

A comparison between the pure crystalline form of actinomycin D (Sigma) and a more soluble clinical form (Cosmegen) was also conducted. No differences could be detected as is shown below:

After 0.01 mg there were 4.3 (Cosmegen) and 4.6 (Sigma) apoptotic cells per crypt section at 6 h, and after 0.1 mg 7.5 (Cosmegen) and 7.3 (Sigma). $X_{med}$ and $\sigma_r$ before crypt size correction, when expressed as ($X_{med}$, $\sigma_r$), were (7.1, 4.7) (Cosmegen) and (7.2, 4.2) (Sigma) after 0.01 mg, and (6.4, 4.2) (Cosmegen) and (6.6, 4.5) (Sigma) after 0.1 mg. After crypt size correction all the $X_{med}$ values lay within the range of 7.2–7.7, and the $\sigma_r$ values were within the range 4.5–5.2. The number of mitotic figures per crypt section 6 h after treatment were also similar: 1.3 (Cosmegen) and 1.4 (Sigma) after 0.01 mg, and 0.5 for both sources of ACT after 0.1 mg.

**Figure 7** An illustration of the scatter range in the parameters for the apoptotic distributions. The upper three panels show the points ($X_{med}$, $\sigma_r$) of 16 individual mice for each of three treatments, i.e. $\gamma$-ray (0.5 Gy, 3 h), CP (5 mg, 6 h) and HU (1 mg, 3 h) repeated as four separate experiments conducted on each of four successive weeks. Fifty half-crypts were scored from each mouse. The parameters for the pooled data from 4 mice (i.e. 200 half-crypts) are shown by the solid circles in the lower panels. The parameters from the distribution for all 16 mice pooled (800 half-crypts) are shown by a double circle. See **Appendix A**.

**Figure 8** Comparison of the median values of the distributions obtained by two different scorers. The same samples as in **Figure** 7 were counted by two scorers A and B. Four points, each being from 4 mice (solid circle) and one point for the pooled data from 16 mice (double circle) are shown for three treatments, i.e. $\gamma$-ray (0.5 Gy, 3 h), CP (5 mg, 6 h) and HU (1 mg, 3 h). The straight line at 45° shows the agreement between the two scores.

### Appendix B

*Points on the distribution used for velocity calculation*

The median ($X_{med}$) and a measure of the spread of the distribution ($\sigma_r$ and $\sigma_l$) are defined mathematically elsewhere (Ijiri & Potten, 1983) and can be used to characterise the apoptotic distributions. The points $X_{med}$, $X_{med}+0.674\sigma_r$ and $X_{med}+1.177\sigma_r$ (Figure 2d) are employed in the present study to calculate the velocity. Although these are merely arbitrary points on the distribution, the displacement of which with time can be measured, some arguments can be put for using these parameters. The median was adopted because some distributions were skewed, in which case $X_{med}$ represents the central value (the peak point) better than the mean. (see **Appendix** in the previous paper, Ijiri & Potten, 1983). Because the peak value (mode) on the actual distribution tends to fluctuate more than the median the latter was again preferred. $X_{med}+0.674\sigma_r$ and $X_{med}+1.177\sigma_r$ represent points on the distribution at 3/4 of the total distribution and the half peak value respectively (Ijiri & Potten, 1983; Kaur & Potten, 1986).

The overall cell migration pattern in the crypt is complicated with one pathway from the stem cells (at about cell position 4) to the upper cell positions and another slower pathway from the stem cells downwards to the Paneth cells. Since all the median values for various cytotoxic agents were found to be more than 4, it can be expected that the cell positions to the left of the median contain all, or most, of the Paneth cells and this is not true for the positions to the right of the median. In this paper the migration velocity of apoptotic fragments is calculated from the displacement of the right half of the distribution up the crypt with increasing time, i.e. only the upward migration has been considered. On the assumption that the right half is half of a normal distribution with the median as its peak and $\sigma_r$ as its standard deviation, the positions of the upper 1/4 (the upper quartile) of the total distribution, and the half-peak position were theoretically given by $X_{med}+0.674\sigma_r$ and $X_{med}+1.177\sigma_r$, respectively (Figure 2d). Having put $z=(X-X_{med})/\sigma_r$, the following standardised normal curve was considered: $f(z)=(1/\sqrt{2\pi})e^{-z^2/2}$. The figure 0.674 was obtained when the area under the normal curve to the right of this point is 25% of the total population, i.e. $P(z\geq 0.674)=0.25$ or $P(0\leq Z<0.674)=P(Z\geq 0.674)$. The half-peak position was obtained solving the following equation: $f(z)=f(0)/2$, which gave us $z=2\sqrt{\ln 2}=1.177$. The movement of the half-peak position has been used by others to assess the migration velocity of the [$^3$H]TdR-labelled cells in the crypts of rats and mice (Cairnie *et al.*, 1965; Wright *et al.*. 1974; Potten *et al.*, 1982; Kaur & Potten, 1986).

When the regression line is fitted to values of $X_{med}(t)$ over the time interval from $t=t_1$ to $t=t_2$, $v$ expresses the average velocity over the cell positions between $X_{med}(t_1)$ and $X_{med}(t_2)$, which corresponds to the time interval between $t_1$ and $t_2$. An average cell position can be defined as $X_{med}((t_1+t_2)/2)$ as indicated by the letter B in Figure 2c, and the value $v$ can be plotted as the velocity at this average position (shown in Figure 9). Here, three points on the apoptotic distribution, i.e. $X_{med}$, $X_{med}+0.674\sigma_r$ and $X_{med}+1.177\sigma_r$ were chosen (Figure 2d). The velocities of the points $X_{med}+0.674\sigma_r$ and $X_{med}+1.177\sigma_r$ were calculated in a similar manner as outlined above for $X_{med}$.

*Migration velocity of apoptotic fragments in crypts treated with cytotoxic agents*

Some apoptotic fragments will be engulfed by neighbouring healthy cells which if themselves in motion will carry the fragment up the crypt. The velocity of such apoptotic fragments after the administration of each cytotoxic agent was evaluated for each dose, calculating the movement of three arbitrary points on the distribution, namely $X_{med}$, $X_{med}+0.674\sigma_r$ and $X_{med}+1.177\sigma_r$ after crypt size correction. The position of each of these points can then be plotted against time after treatment and a regression line then can be fitted (see Figures 2c and 2d). The dose and agent used for these calculations are listed in the right-hand column of Table I and marked with a +. In total, 28 doses of 18 agents were analysed. Since three different points on the distribution were analysed a total of 84 velocities estimates were obtained and are plotted in Figure 9. Since the 84 data points are from 18 different cytotoxic agents, whose modes of action, action time and levels of damage differ, the data for each agent should really be looked at separately. However, many unknown factors are involved and for the moment all 84 points will be considered together. The data covered most of the cell positions in the crypt. The datum points were widely scattered but were mostly within the range 0–0.5 cell positions $h^{-1}$, with a tendency to increase with increasing cell position.

The data were subjected to a simple linear regression using least squares. The resulting equation was $v=0.0118x+0.0667$, where $v$ is the velocity of migration (cell positions $h^{-1}$) and $x$ the cell position. The residual mean squares had a value of 0.0333, and the velocity, with its 95% confidence interval at several cell positions, was as follows: $0.126\pm0.022$ (cell positions $h^{-1}$) at cell position 5, $0.173\pm0.020$ at cell position 9, $0.206\pm0.020$ at position 11.8 (where the confidence limit is minimal), $0.220\pm0.020$ at position 13, and $0.315\pm0.025$ at position 21. As expected from the scattering of the data, the coefficient of determination was low at $r^2=0.046$.

The migration velocity of apoptotic fragments (Figure 9) indicated that there was a gradual shift to the right in the distributions of apoptotic cells with time. This occurred in spite of the severe cytotoxic damage, i.e. under conditions of reduced numbers of cells in the crypt and reduced cell division activity. The apoptotic fragments move probably because some are ingested by neighbouring healthy cells, which themselves move up the crypt and onto the villus. However, the velocities calculated here tend to be lower than those calculated using [$^3$H]TdR labelled cells. This is probably due to the fact that: (1) some of the cytotoxic agents may affect directly the mechanism involved in cell movement. (2) not all apoptotic fragments are incorporated into migrating epithelial cells, e.g. those engulfed by Paneth or stem cells or those lost to the crypt lumen. (3) the general damage caused to the crypt by the cytotoxic agents may result in severe structural damage to the crypt cells (including peripheral fibroblast cells) so that cells can no longer migrate normally.

For some agents, the velocity of the highest cell position with apoptotic fragments (the leading edge of the distribution) was estimated, using the 99th percentile point (i.e. the upper 1% point of the distribution) in crypts where the size correction had been applied. A regression line was then fitted as described in Figure 2 and the velocities were calculated. These were, HU (1.2 cells $h^{-1}$, at cell position 26) and CP (0.9, at cell position 23). The data for IMS-treated and $\gamma$-ray-treated animals were 0.6 cells $h^{-1}$ (at cell position 22) and 0.5 (at 20) respectively. These are similar to those reported

**Figure 9** Migration velocity of apoptotic fragments at different cell positions along the crypt column treated with various cytotoxic agents. Eighty-four velocity values for various cell positions after treatment with 18 different agents are plotted. For each dose level of an agent, the movement of three points on the distribution, i.e. $X_{med}$, $X_{med}+0.674\sigma_r$ and $X_{med}+1.177\sigma_r$ was traced with time and the migration velocities were determined as described in **Figures 2c and 2d**. Thus, there are 3 or 6 points for each agent (one or two dose levels). The abscissa (cell position) gives the average cell position determined as B in **Figure 2c** for $X_{med}$, and in a similar manner for other two points on the distribution. A simple linear regression was fitted to the velocity ($v$, cell positions $h^{-1}$) *vs.* cell position ($x$), resulting in an equation $v=0.0118x+0.0667$ with its 95% confidence limit being within a difference of $\pm0.025$ from the line over cell positions 5 to 21. The deviation of individual data from the regression line was $=0.183$. Symbols as for **Figures 4 and 5**.

for normal mice using the leading edge of the distributions of [$^3$H]TdR-labelled cells: 0.7 cell positions $h^{-1}$ at cell positions 18–25 (Potten *et al.*, 1982), and 1.05 cell positions $h^{-1}$ at cell position 24 (Cheng & Leblond, 1974) and $\sim$1.5 cell positions $h^{-1}$ at cell position 20 (Kaur & Potten, 1986). These velocity estimates suggest that those cells unaffected by the cytotoxic insult, near the top of the crypt, continue to move in a near normal manner carrying any engulfed fragments with them. This results in a broadening of the distributions of apoptotic fragments (e.g. a broadening of $\sigma_r$) with the passage of time.

VCR arrests cells in mitosis some of which will die and degenerate and may appear pycnotic. No distinction has been made here between pycnosis of arrested mitotic figures and apoptosis. Occasionally it was difficult to distinguish arrested mitotic figures from pycnotic mitotic figures but subjective criteria were established to facilitate a discrimination between these two classes of mitotic cells. Some clearly apoptotic cells were also observed as early as 3 h after VCR, some of which were probably the consequence of the direct action of VCR (Figure 3c). This was slightly more prominent after a dose of 0.1 mg. Further differences between the results obtained using two different doses of VCR were seen when various times after treatment were studied. After 0.01 mg the number of mitotic cells decreased with time (from 3 to 12 h), while the number of apoptotic cells increased up to 9 h (probably due to degeneration from the arrested metaphases). The apoptotic yield then decreased (presumably due to the disappearance of the degenerating cells). Similar results have been reported after VCR treatment of mouse ascites tumour cells (Camplejohn *et al.*, 1980). After the higher dose (0.1 mg) the number of mitotic figures remained constant over the period 3–12 h post-treatment, while there was a continuous increase in the number of apoptotic cells (Figure 3c). After the high dose of VCR new metaphases may continue to be arrested even after times greater than 3 h. However, the possibility cannot be excluded that the discrepancy in the results when the two doses are compared may be due to the difficulty in distinguishing mitotic figures from apoptotic cells. For this reason the combined distributions of mitotic figures and apoptotic fragments were analysed for the study on cell movement (the leading edge). For 0.01 mg VCR this was 0.7 cells $h^{-1}$ at cell position 26, and for 0.1 mg VCR, 0.5 cells $h^{-1}$ at cell position 26.

### References

CAIRNIE, A.B., LAMERTON, L.F. & STEEL, G.G. (1965). Cell proliferation studies in the intestinal epithelium of the rat. I. Determination of the kinetic parameters. *Expl. Cell Res.*, **39**, 528.

CAMPLEJOHN, R.S., SCHULTZE, B. & MAURER, W. (1980). An *in vivo* double labelling study of the subsequent fate of cells arrested in metaphase by vincristine in the JB-1 mouse ascites tumour. *Cell Tissue Kinet.*, **13**, 239.

CHENG, H. & LEBLOND, C.P. (1974). Origin, differentiation and renewal of the four main epithelial cell types in the mouse small intestine. I. Columnar cell. *Am. J. Anat.*, **141**, 461.

HENDRY, J.H., MOORE, J.V. & POTTEN, C.S. (1984). The proliferative status of microcolony-forming cells in mouse small intestine. *Cell Tissue Kinet.*, **17**, 41.

HENDRY, J.H. & POTTEN, C.S. (1982). Intestinal cell radiosensitivity: a comparison for cell death assayed by apoptosis or by a loss of clonogenicity. *Int. J. Radiat. Biol.*, **42**, 621.

HUME, S.P., MARIGOLD, J.C.L. & FIELD, S.B. (1979). The effect of local hyperthermia on the small intestine of the mouse. *Br. J. Cancer*, **52**, 657.

IJIRI, K. & POTTEN, C.S. (1983). Response of intestinal cells of differing topographical and hierarchical status to ten cytotoxic drugs and five sources of radiation. *Br. J. Cancer*, **47**, 175.

IJIRI, K. & POTTEN, C.S. (1984). The re-establishment of hypersensitive cells in the crypts of irradiated mouse intestine. *Int. J. Radiat. Biol.*, **46**, 609.

IJIRI, K. & POTTEN, C.S. (1986). Radiation-hypersensitive cells in small intestinal crypts; their relationships to clonogenic cells. *Br. J. Cancer*, **53**, Suppl. VII, 20.

IJIRI, K. & POTTEN, C.S. (1987). Cell death in cell hierarchies in adult mammalian tissues. In *Perspectives on Mammalian Cell Death*, Potten, C.S. (ed) Chap. 13. Oxford Univ. Press (in press).

KAUR, P. & POTTEN, C.S. (1986). Cell migration velocities in the crypt after cytotoxic insult are not dependent on mitotic activity. *Cell Tissue Kinet.*, **19**, 601.

MEISTRICH, M.L., FINCH, M., DA CUNHA, M.F., HACKER, U. & AU, W.W. (1982). Damaging effects of fourteen chemotherapeutic drugs on mouse testis cells. *Cancer Res.*, **42**, 122.

MOORE, J.V. (1979). Ablation of murine jejunal crypts by alkylating agents. *Br. J. Cancer*, **39**, 175.

MOORE, J.V. (1985). Clonogenic response of cells of murine intestinal crypts to 12 cytotoxic drugs. *Cancer Chemother. Pharmacol.*, **15**, 11.

POTTEN, C.S. (1977). Extreme sensitivity of some intestinal crypt cells to X- and γ-irradiation. *Nature*, **269**, 518.

POTTEN, C.S. (1983). Stem cells in gastrointestinal mucosa. In *Tumor Viruses and Differentiation*, Scolnick & Levine (eds) p. 381. Alan R. Liss: New York.

POTTEN, C.S. & HENDRY, J.H. (1975). Differential regeneration of intestinal proliferative cells and cryptogenic cells after irradiation. *Int. J. Radiat. Biol.*, **27**, 413.

POTTEN, C.S. & HENDRY, J.H. (1983). Stem cells in murine small intestine. In *Stem Cells: Their Identification and Characterisation*, Potten, C.S. (ed) p. 155. Churchill Livingstone: Edinburgh.

POTTEN, C.S., AL-BARWARI, S.E., HUME, W.J. & SEARLE, J. (1977). Circadian rhythms of presumptive stem cells in three different epithelia of the mouse. *Cell Tissue Kinet.*, **10**, 557.

POTTEN, C.S., CHWALINSKI, S., SWINDELL, R. & PALMER, M. (1982). The spatial organization of the hierarchical proliferative cells of the crypts of the small intestine into clusters of synchronized cells. *Cell Tissue Kinet.*, **15**, 351.

SEARLE, J., LAWSON, T.A., ABBOTT, P.J., HARMON, B. & KERR, J.F.R. (1975). An electron-microscope study of the mode of cell death induced by cancer-chemotherapeutic agents in populations of proliferating normal and neoplastic cells. *J. Pathol.*, **116**, 129.

SIGDESTAD, C.P., BAUMAN, J. & LESHER, S.W. (1969). Diurnal fluctuations in the numbers of cells in mitosis and DNA synthesis in the jejunum of the mouse. *Expl. Cell Res.*, **58**, 159.

WITHERS, H.R. & ELKIND, M.M. (1970). Microcolony survival assay for cells of mouse intestinal mucosa exposed to radiation. *Int. J. Radiat. Biol.*, **17**, 261.

WRIGHT, N.A., WATSON, A.J., MORLEY, A.R., APPLETON, D.R., MARKS, J. & DOUGLAS, A. (1974). The measurement of cell production rates in the crypts of Lieberkuhn. An experimental and clinical study. *Virchows Arch. A.*, (*Pathol., Anat.*), **364**, 311.

WYLLIE, A.H. (1981). Cell death: a new classification separating apoptosis from necrosis. In *Cell Death in Biology and Pathology*, Bowen & Lockshin (eds) p. 9. Chapman & Hall: London.